# Measuring Intratumoral Heterogeneity of Immune Repertoires

**DOI:** 10.3389/fonc.2020.00512

**Published:** 2020-05-08

**Authors:** Diana Vladimirovna Yuzhakova, Lilia N. Volchkova, Mikhail Valerievich Pogorelyy, Ekaterina O. Serebrovskaya, Irina A. Shagina, Ekaterina A. Bryushkova, Tatiana O. Nakonechnaya, Anna V. Izosimova, Daria S. Zavyalova, Maria M. Karabut, Mark Izraelson, Igor V. Samoylenko, Vladimir E. Zagainov, Dmitriy M. Chudakov, Elena V. Zagaynova, George Vladimirovich Sharonov

**Affiliations:** ^1^Laboratory of Genomics of Antitumor Adaptive Immunity, Privolzhsky Research Medical University, Nizhny Novgorod, Russia; ^2^Genomics of Adaptive Immunity Department, Shemyakin and Ovchinnikov Institute of Bioorganic Chemistry, Moscow, Russia; ^3^Department of Molecular Technologies, Institute of Translational Medicine, Pirogov Russian National Research Medical University, Moscow, Russia; ^4^Department of Molecular Biology, Moscow State University, Moscow, Russia; ^5^Oncodermatology Department, N. N. Blokhin Russian Cancer Research Center, Moscow, Russia; ^6^Volga District Medical Centre Under Federal Medical and Biological Agency, Nizhny Novgorod, Russia; ^7^Adaptive Immunity Group, Central European Institute of Technology, Masaryk University, Brno, Czechia; ^8^MiLaboratory LLC, Skolkovo Innovation Centre, Moscow, Russia

**Keywords:** tumour heterogeneity, clonal expansions, tumour clonality, TCR repertoire, immunoglobulin repertoire

## Abstract

There is considerable clinical and fundamental value in measuring the clonal heterogeneity of T and B cell expansions in tumors and tumor-associated lymphoid structures—along with the associated heterogeneity of the tumor neoantigen landscape—but such analyses remain challenging to perform. Here, we propose a straightforward approach to analyze the heterogeneity of immune repertoires between different tissue sections in a quantitative and controlled way, based on a beta-binomial noise model trained on control replicates obtained at the level of single-cell suspensions. This approach allows to identify local clonal expansions with high accuracy. We reveal *in situ* proliferation of clonal T cells in a mouse model of melanoma, and analyze heterogeneity of immunoglobulin repertoires between sections of a metastatically-infiltrated lymph node in human melanoma and primary human colon tumor. On the latter example, we demonstrate the importance of training the noise model on datasets with depth and content that is comparable to the samples being studied. Altogether, we describe here the crucial basic instrumentarium needed to facilitate proper experimental setup planning in the rapidly evolving field of intratumoral immune repertoires, from the wet lab to bioinformatics analysis.

## Introduction

Analysis of T and B cell repertoires has become a valuable and powerful tool for characterizing the immune response, complementing other high-content approaches such as transcriptome analysis and mass cytometry (1–3). Both T and B cell repertoires have been shown to be predictive of survival for cancer patients (4–8) and for assessing response to checkpoint immunotherapy (9–13).

Immune repertoires allow for tracking lymphocyte lineages, and make it possible to trace the evolution and heterogeneity of anti-tumor immunity. For example, immune repertoire analysis has been used to reveal that most tumor-infiltrating regulatory T cells in human cancers originate in the thymus rather than from local conversion of conventional T cells (14–16). Tumeh et al. have shown that pre-existing tumor-infiltrating CD8^+^ T cell clones expand after PD-1 checkpoint blockade and this underlies the positive response of advanced melanoma patients to therapy (10). In contrast, more recent study of basal and squamous cell carcinoma treated with anti-PD-1 revealed clonal expansion of novel clonotypes that had not previously been observed in the tumor, but not of pre-existing tumor-infiltrating lymphocytes (TILs) (17). Based on limited available information on T cell repertoires, Zhao et al. have suggested that there is increased clonality (i.e., decreased diversity) of TILs after anti-PD-1 therapy in treatment-responsive cases of glioblastoma and decreased clonality (i.e., increased diversity) in non-responders (18). On the other hand, Schalper et al., made the determination based on (also limited) TCR repertoire information obtained using multiplex PCR from formalin-fixed paraffin-embedded (FFPE) block-extracted RNA that a decrease in T cell clonality in glioblastoma patients receiving the same treatment was associated with longer survival (19).

In one of the first deep sequencing-based efforts to estimate intratumoral T cell receptor (TCR) repertoire heterogeneity, Emerson and colleagues reported relatively high TCR repertoire similarity throughout ovarian carcinoma tumors in terms of clonal overlap (20). At the same time, in melanoma, it has been shown that TILs harvested from different tumor fragments possess different reactivity against melanoma-associated antigens, and that corresponding epitopes were typically found in the same tumor fragments as their cognate TCRs (21, 22), although these clones were present at low frequencies (21). Subsequent studies have revealed intratumoral heterogeneity of T and B cells in lung adenocarcinoma (6, 23, 24), esophageal squamous cell carcinoma (25, 26), colorectal (27), ovarian (28), breast (29), and pancreatic cancers (30).

In several recent studies, immune repertoires were analyzed in conjunction with genomic heterogeneity of tumor cells. These data revealed that immune surveillance evolves with the tumor, with certain T cell clones tracking tumor neoantigens both spatially and temporally and imposing selection pressure on the tumor (6, 23, 24, 27, 28). For B cells, the affinity maturation of immunoglobulins against tumor antigens was deduced from repertoire analysis (5, 7, 31), although no association with tumor clonality and evolution has been revealed to date.

One of the main complications in comparing immune repertoires between different timepoints and distinct tumor sections is that there is always some dispersion in clonal frequencies caused by sampling limitations. These can arise at the level of tissue sampling, T or B cell counts, the amount and quality of extracted genomic DNA (gDNA) or RNA, efficiency of cDNA synthesis and template molecules entrance into PCR amplification. The stochastic nature of PCR amplification further adds artificial dispersion. RNA-based TCR and immunoglobulin repertoire profiling is particularly informative in terms of assessing the functional activity of infiltrating T and B cells and local antibody production, especially given the ultra-high immunoglobulin expression levels seen in plasma cells. However, in such experiments, the cell-to-cell dispersion in expression levels can further increase the artificial repertoire heterogeneity originating from one or more randomly-sampled or under-sampled plasma cells or active effector T cells. These sources of natural and technical noise have to be taken into account and distinguished from actual repertoire differences.

Some of the aforementioned works employed replicates produced at the level of split gDNA samples. This potentially allowed the authors to control for the dispersion in TCR or BCR repertoire content arising from technical errors (20, 25, 26, 30), with the exception of sample-to-sample heterogeneities associated with the DNA extraction procedure and thus, importantly, differences in sampling depth. However, this information has not been implemented to build an appropriate noise model.

Here, we propose a straightforward approach for measuring the extent of TCR and immunoglobulin heterogeneity in tumor samples relative to internal controls in terms of baseline repertoire dispersion. This is estimated using tissue sample replicates that have been split at the level of homogenized cells. These replicates are then used to train a beta-binomial noise model, as suggested by Rytlewski et al., which is further used to exclude false positives amongst differentially represented clones (32).

We use this approach to demonstrate uneven clonal distribution of CD8^+^ T cells in poorly-infiltrated B16F0-derived tumors in a mouse model of melanoma (33). This is associated with clustered distribution of these cells within the tumor, based on multicolor fluorescent immunohistochemistry analysis, indicating local proliferation of CD8^+^ T cells *in situ* at the tumor site. Working with human tissue samples, we also demonstrate heterogeneous distribution of plasma cell clones in a lymph node heavily infiltrated by metastatic melanoma and in a primary colorectal tumor. We also show a scenario in which training with high quality, deeply-analyzed biological replicates may lead to identification of false-positive clonal expansions when analyzing more noisy samples of interest. This highlights the importance of replicas for correct repertoire comparison, and of careful use of this analytical tool.

## Results

### Lymphocyte Infiltration Pattern of B16F0 Melanoma

**Figure 1:**
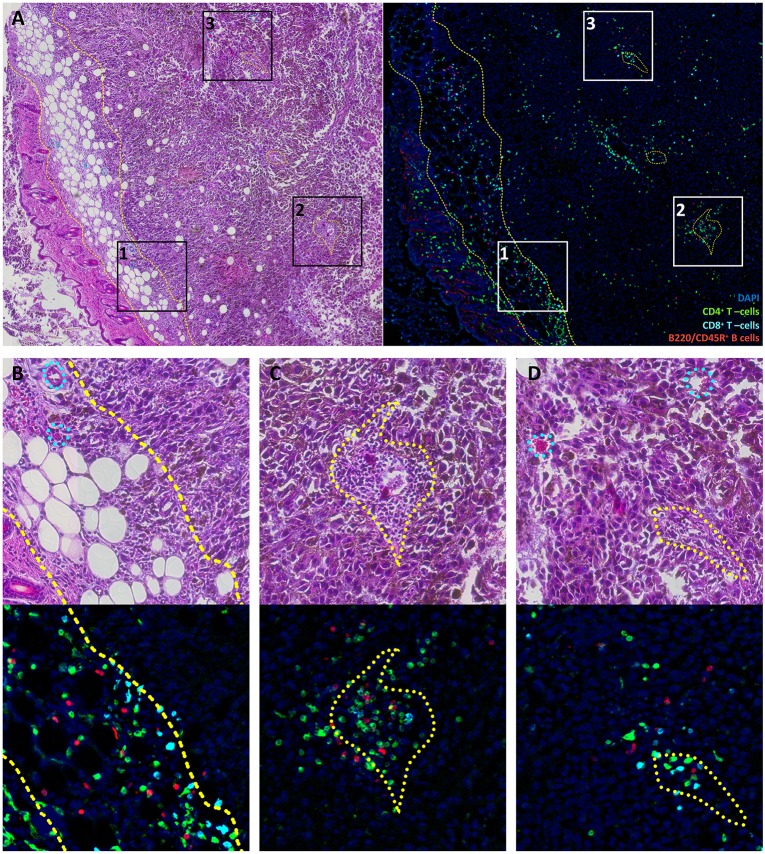
Lymphocyte distribution in B16F0 mouse melanoma. **(A)** Overview image of the tumor and surrounding tissue labeled with H&E staining (left) or multicolor immunofluorescence (right). **(B–D)** show close-up of rectangles **1**, **2**, and **3** from panel **(A)**. Green represents CD4^+^ T cells, cyan represents CD8^+^ T cells, red represents B220/CD45R^+^ B cells and blue indicates DAPI-stained nuclei. Yellow dashed curves outline subcutaneous fibrous tissue that constitutes the tumor capsule. Yellow dotted curves outline regions that surround vessel and are enriched in leukocytes. Cyan dotted curves on H&E images show blood vessels and capillaries that have no prominent leukocyte pockets. It should be noted that tissue structures are marked based on H&E images; these marks do not coincide directly with cells in the fluorescence images since these show different slices spaced ~20 μm apart.

The spatial clonal heterogeneity of TILs has not been thoroughly studied in mouse tumor models, and it is an intriguing question whether such heterogeneity exists and how it can affect repertoire-based analysis. Uncovering such heterogeneity could also shed light on sources of TILs for corresponding models. In order to reveal possible sources of TIL clonal heterogeneity within tumors, we first studied their patterns of distribution in mouse melanoma. Using multicolor IHC, we analyzed the distribution of CD4^+^/CD8^+^ T cells and B cells in whole tumor tissue slices from a B16F0 melanoma model. We found a common distribution pattern for all lymphocyte subsets, with prominent accumulation in the fibrous tumor capsule and in several large clusters within tumor nodes (Figure 1). The tumor capsule is characterized by a high density of immature, hyper-permeable blood vessels that facilitate lymphocyte infiltration (34), while surrounding loose connective tissue offers a perfect substrate for further lymphocyte migration (35). This may result in relatively non-specific lymphocyte accumulation in the surrounding tumor envelope. Prior work has also shown that T cells in tumor nodes are more clonal and associated with lower clonal diversity compared to stromal T cells in ovarian tumors (28).

Lymphocyte clusters within the tumor were also related to certain morphological structures, as revealed by comparison of fluorescently-labeled and histological slices. One common feature of these structures was the presence of a blood vessel within the pocket that almost exclusively contained leukocytes (Figure 1C). It should be noted that only about 25% of blood vessels within the tumor were so prominently surrounded by leukocytes. These are likely to be high endothelial venule pockets that have analogous histological appearance, and give rise to tertiary lymphoid structures (36–38). These intratumoral clusters of CD4^+^ and CD8^+^ T cells may originate from locally enhanced infiltration and/or local proliferation of clonal T cell populations. The latter would be expected to lead to a highly heterogeneous distribution of T cell clones across the tumor.

### Pipeline for Measuring Heterogeneity and Local T Cell Expansion

**Figure 2:**
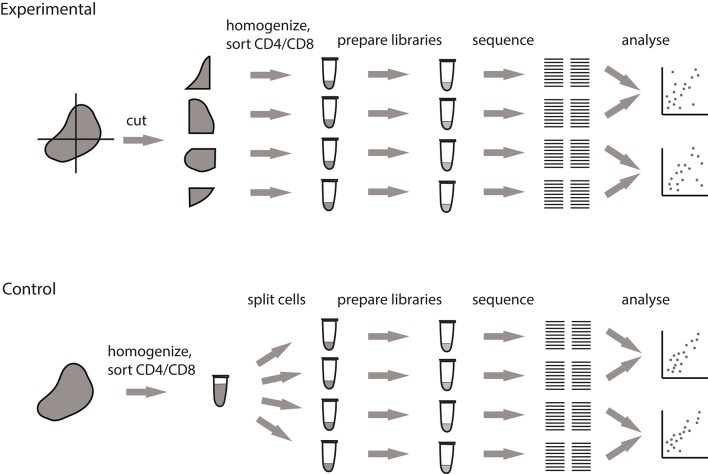
Pipeline for measuring the extent of local intratumoral T cell expansions. Control samples are generated by splitting the replicas at the level of single-cell suspension. Experimental samples are split at the level of tissue fragments.

To clarify the origin of observed clusters, we designed a pipeline that allows to measure the contribution of local clonal expansions to repertoire heterogeneity. This approach fully accounts for natural dispersion in clonal frequencies between samples that originates from sampling limitations and is unrelated to real clonal heterogeneity (Figure 2).

We processed tumor masses using two alternative methods, both of which produce multiple single-cell suspensions. In the first setup (experimental), the tumor is dissected into four fragments of comparable size, and each fragment is processed independently. In the second setup (control), the whole tumor sample is first homogenized and filtered, after which the four replicates are split from the resulting PBS-washed single cell suspension. Further TCR repertoire profiling of these control samples allows to measure the natural sample-to-sample variation resulting from stochastic factors associated with cell sampling and sorting, cell-to-cell variation in TCR mRNA expression, and mRNA and cDNA sampling in the course of library preparation.

In these experiments, we were particularly interested in the nature of intratumorally-observed clusters of T cells. Therefore, we carefully cleaned the excised tumors from the collagenous envelope, including the fibrous tumor capsule shown in Figure 1, in order to focus our analysis on lymphocyte heterogeneity within the tumor parenchyma. It should be noted that our ability to estimate clonal frequencies—and thus the extent of correlation of these frequencies between replicates and distinct tumor sections—is intrinsically limited by sampling bottlenecks and the resulting depth of profiling in terms of cell counts and template gDNA/cDNA counts. Table 1 summarizes the number of sorted cells and unique UMI-labeled TCRβ cDNA molecules analyzed for each sample.

**Table 1:** Cells, molecules and clonotypes in replicates of B16F0-infiltrating T cells.

**Setup**	**Cell subset**	**# T cells sorted**	**# TCRβ UMIs**	**# clonotypes**	Clonality^*^
Experimental	CD4	11,245	3,710	1,417	0.03
Experimental	CD4	6,519	1,737	781	0.03
Experimental	CD4	10,922	6,757	1,967	0.03
Experimental	CD4	8,904	3,015	1,280	0.03
Experimental	CD8	14,270	12,708	1,052	0.24
Experimental	CD8	10,375	12,597	786	0.3
Experimental	CD8	22,436	29,176	1,483	0.31
Experimental	CD8	14,284	17,355	1,030	0.31
Control	CD4	44,232	5,210	2,174	0.03
Control	CD4	25,182	17,836	3,691	0.03
Control	CD4	27,891	10,852	3,004	0.04
Control	CD4	13,022	4,106	1,572	0.04
Control	CD8	20,794	15,361	2,365	0.21
Control	CD8	13,022	3,109	734	0.21
Control	CD8	14,112	13,614	1,800	0.23
Control	CD8	6,494	3,556	693	0.23

* *Clonality metrics [1-Normalized ShannonWiener Index] was calculated as in Tumeh et al. (10) but after down-sampling to an equal number of 500 UMI-labeled TCRβ cDNA molecules (39)*.

### Measuring Intratumoral Clonal Heterogeneity of T Cells

Seven melanoma-bearing mice were randomly subdivided into two groups with average tumor volume of 0.23 ± 0.7 (Mean ± SD) mm^3^ and 0.32 ± 0.11 mm^3^ for control and experimental group, respectively, and processed by either experimental procedure. After removal of outer tumor capsule density of T cell infiltration did not correlate with tumor volume and constituted 280 ± 150 cells per μl of tumor tissue.

**Figure 3:**
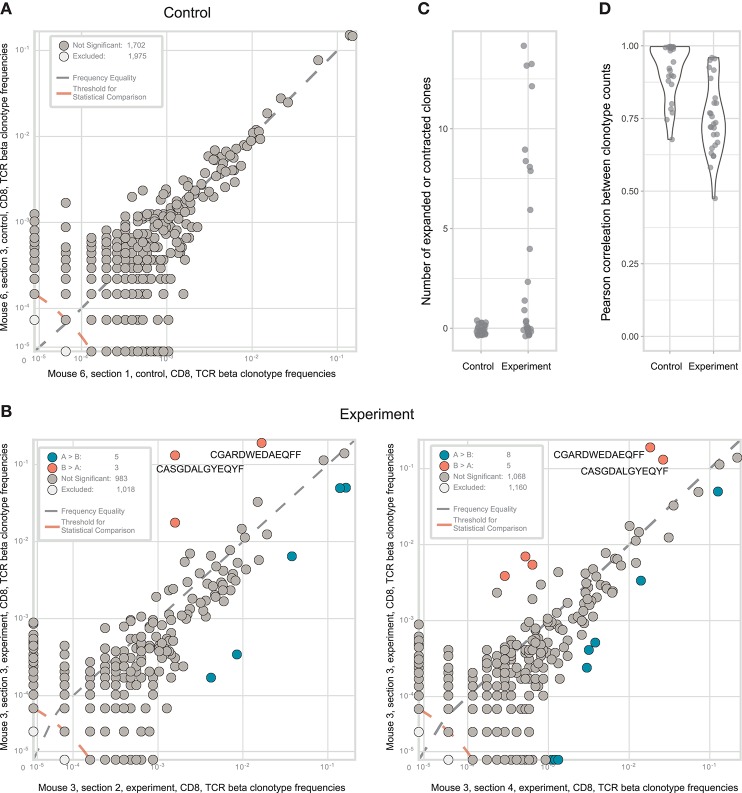
Identification of CD8^+^ T cell clones heterogeneously distributed within tumor samples from a mouse model of melanoma. **(A)**. The concentration of each TCRβ CDR3 clonotype in the first control sample is plotted against the concentration of the same clonotype in the second control sample. **(B)**. The concentration of each TCRβ CDR3 clonotype in tumor section #3 (y-axis) is plotted against the concentration of the same TCR clonotype in tumor sections #2 and #4 (x-axis). Clonotype variants that were identified as significantly expanded in one of the tumor sections are shown in orange or blue. **(C)**. Numbers of expanded or contracted clones between all pairs of repertoires in four experimental and three control tumor. **(D)**. Pearson's *r* measurement of correlation between counts of clones present in each pair of control or experimental repertoires.

We compared the correlation of clonal TCRβ frequencies for the replicates obtained using both setups. As expected, clonal frequencies were highly correlated between biological replicates in control tumor sections (Figure 3A, see https://figshare.com/s/3e89769057700942f6cd for all correlation scatterplots). The most abundant clones were of comparable frequency in all replicates. At the same time, this correlation was not ideal, and the extent of this disparity reflected the natural dispersion between hypothetically “identical” tumor replicates due to sampling effects and stochasticity during library preparation. In contrast, correlation of clonal frequencies between experimental tumor sections was significantly lower (Mann Whitney *U*-test, *p* < 0.0001; Figures 3B,C). This poorer correlation compared to the control method reflects the true heterogeneity between the analyzed samples, accounting for all of the technical limitations and bottlenecks.

In some of the experimental tumor sections, particular CD8^+^ clones showed drastic expansion (Figure 3B). For example, the clonal TCRβ CDR3 variant CGARDWEDAEQFF occupied ~19% of the repertoire in tumor section #3 but <3% in the other sections. Similarly, CDR3 variant CASGDALGYEQYF occupied ~13% of the repertoire in section #3 vs. just 0.1–1.8% in the other sections. To statistically identify clonotypes that are differentially expanded across two tumor sections, we used an approach suggested by Rytlewski et al., in which a pair of control repertoires with medium numbers of clones was used to train a beta-binomial noise model. Using this model, we identified significantly contracted and expanded clones between each pair of repertoires (Figure 3C; Mann-Whitney *U*-test, *p* = 0.0001). Notably, such clones were only found in experimental samples, and never between control replicate samples. These results were stably reproducible in three control and four experimental tumor preparations (Figures 3C,D). Based on these findings, we concluded that the observed intratumoral clusters of CD8^+^ T cells (Figure 1) are probably formed by progeny of T cells that infiltrated the tumor via high endothelial venules, but then proliferated *in situ* to form relatively large local clonal expansions.

It must also be noted that such local clonal expansions were consistently identified almost exclusively among CD8^+^ but not among CD4^+^ T cells. In general, CD4^+^ T cells rarely form expansions as large as those formed by CD8^+^ T cells, reflected by lower clonality score (see Table 1). Thus, we believe that the sensitivity and accuracy of repertoire heterogeneity analysis is insufficient to reliably assess relatively minor clonal expansions that would be expected to intrinsically occur amongst CD4^+^ T cells. Analysis of larger lymphoid structures [e.g., tertiary lymphoid structures in human cancers (38)] should reveal statistically significant local CD4^+^ expansions.

### Measuring Intratumoral Clonal Heterogeneity of B Cells

Heterogeneity of immunoglobulin repertoires across tumor tissues can be investigated in a similar fashion, with the caveat that immunoglobulin expression levels differ dramatically between naive, memory, and plasma B cells by an average ratio of ~2:5:500 (40), with high dispersion between B cell clones and individual cells. The RNA-based analysis of immunoglobulin repertoires mainly provides functional information on the relative abundance of locally produced clonal antibody variants and the most activated effector B cell receptor (BCR) distributions—mixed together, if functional B cell subsets were not sorted first. Thus, questions related to clonal composition of infiltrating B cells—irrespective of their functional activity—should be preferably studied with DNA-based immunoglobulin profiling.

**Figure 4:**
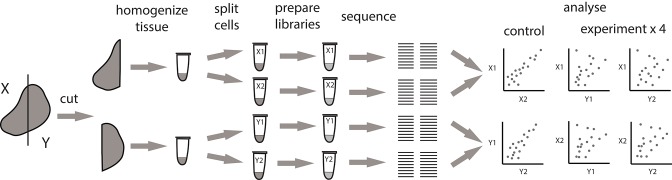
Alternative pipeline for measuring the extent of local intratumoral expansions. Experimental samples are split at the level tissue fragments. Each experimental fragment is further split into replicas at the level of single-cell suspension.

Here, we studied - at the RNA level - immunoglobulin heterogeneity in a sample from the metastatically-infiltrated lymph node of a patient with cutaneous melanoma. We used a modified experimental scheme that compares two tissue sections, with the advantage of having two internal controls and four independent experimental comparisons (Figure 4). According to flow cytometry analysis, CD45^+^ leukocytes constituted 17.7 and 4.8% of all cells in analyzed fragments, of which 28 and 25% were CD19^+^ B cells, respectively. CD20-CD38^+^ plasma cells constituted 11.5 and 21.6% of all B-cells. Fragments were comparable in size, and the number of cells we isolated was also comparable (7,500 and 4,700 plasma cells in total).

**Figure 5:**
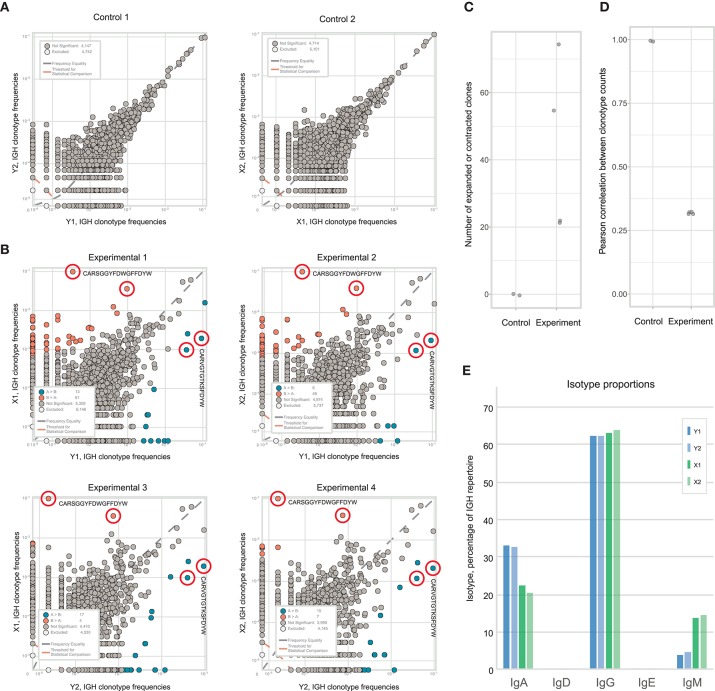
Estimating immunoglobulin repertoire heterogeneity within a melanoma-infiltrated lymph node. **(A)** The concentration of each IGH CDR3 clonotype in one part of fragment is plotted against the concentration of the same clonotype in the other part of that fragment after being split at the level of homogenized cells. **(B)** Pairwise comparison of the four repertoires obtained from two fragments of the tumor. Red circles indicate clonotypes independently identified as expanded in all four comparisons. Blue color shows clonotypes expanded in tumor fragment Y, orange—in fragment X. **(C)** Number of expanded or contracted clones between pairs of control or experimental repertoires. **(D)** Pearson's *r* measurement of correlation between counts of clones present in each pair of control or experimental repertoires. **(E)** Isotype proportions in replicates of the two analyzed fragments.

As expected, clonal frequencies correlated well between replicates from the same section (Figure 5A). In contrast, samples from different tumor fragments showed much lower correlation, as we observed for TCR repertoires from murine tumors. For example, the IGH CDR3 clonal variant CARSGGYFDWGFFDYW occupied 9.9% of the repertoire in tumor fragment X, but represents <0.1% in fragment Y. Likewise, clonal variant CARVGTGTKSFDYW occupied 9% of the repertoire in fragment Y and only 3.5% in fragment X (Figure 5B). Clonal expansions were identified only when comparing the samples obtained from X and Y fragments (Figure 5C), and lower correlation of clonal frequencies between X and Y fragments compared to replicate samples allowed to estimate the level of immunoglobulin heterogeneity (Figure 5D).

Along with the composition, clonality, and hypermutation of intratumorally-produced antibodies, the proportion of distinct antibody isotypes may also be a crucial parameter with prominent prognostic value, as has been shown for human melanoma and subtypes of lung adenocarcinoma and bladder cancer (5, 8). Like clonal frequencies, isotype proportions are heterogeneous across tumor tissue and subject to sampling noise, but also generally correlate well between replicates produced at the level of single-cell suspensions (Figure 5E). Thus, the findings above are equally applicable to the study of the heterogeneity of the isotypic composition of antibodies produced in tumor tissues and the correlation of this heterogeneity with unevenness of the immune landscape.

### Noise Models Must Be Trained on Datasets of Comparable Depth

We next repeated the whole pipeline to assess immunoglobulin heterogeneity in human colon cancer sections, using the same experimental design shown in Figure 4. According to flow cytometry estimates CD45^+^ leukocytes constituted 35–45% of all cells in analyzed section and consisted predominantly from lymphocytes (70–80%), that in turn contained 20–40% of CD19^+^ B cells. Remarkably, both sections contained unusually high proportions of CD19^+^CD20^−^CD38^+^ plasma B cells among all CD19^+^ B cells, on the order of ~50%, suggesting the presence of tertiary lymphoid structures (38). It is also important to note here that in spite of similar volume of the two sections, the total number of isolated cells was significantly different (~1 million vs. 10 million). As a result the two sections contained very different numbers of CD19^+^ B cells and plasma cells, with about 50,000 in fragment X and about 500,000 in fragment Y (Table 2).

**Table 2:** Immunoglobulin repertoires from melanoma lymph node samples and colon cancer samples.

**Sample**	**Tumor section**	**Estimated number of CD19^**+**^CD20^**+**^CD38^**−**^ B cells**	**Estimated number of CD19^**+**^CD20^**−**^CD38^**+**^ plasma cells**	**# IGH UMIs analyzed**	**# IGH CDR3 clonotypes**
Melanoma LN P1	X1	36,000	4,100	144,104	7,167
Melanoma LN P1	X2	36,000	4,100	42,724	4,052
Melanoma LN P1	Y1	22,000	2,200	94,752	6,966
Melanoma LN P1	Y2	22,000	2,200	60,245	5,895
Colon cancer P2	X1	12,000	10,000	270,372	19,759
Colon cancer P2	X2	18,000	17,000	191,923	15,069
Colon cancer P2	Y1	59,000	52,000	189,292	20,267
Colon cancer P2	Y2	140,000	130,000	213,809	23,440

**Figure 6:**
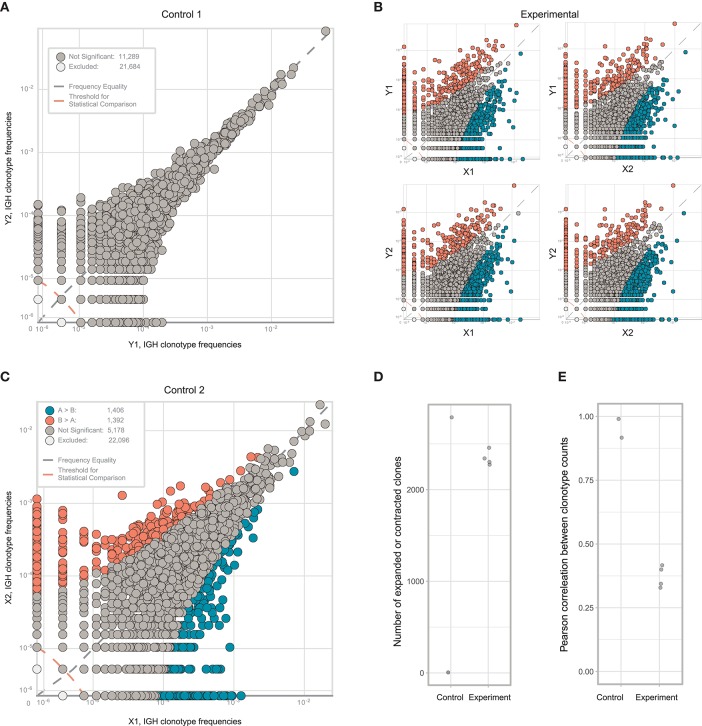
Estimating immunoglobulin repertoire heterogeneity between two sections of colon cancer tissue. **(A)** Frequencies of IGH CDR3 clonotypes in the Y1 and Y2 replicates, which were split at the level of homogenized cells. **(B)** Pairwise comparison of the four experimental repertoires obtained from two fragments and two replicates. **(C)** Frequencies of each IGH CDR3 clonotype in X1 and X2 replicates, which were split at the level of homogenized cells. Orange and blue circles show clonotypes that were erroneously identified as expanded between the two replicates, using a beta-binomial noise model trained on the replicates of fragment Y. **(D)** The number of expanded or contracted clones between control and experimental pairs of repertoires. **(E)** Pearson's *r* correlation between counts of clones present in each control and experimental pair of repertoires.

Both replicates from fragment Y contained high numbers of plasma cells, providing excellent statistics for the accurate identification of clonal frequencies (Figure 6A). We trained the noise model on these replicates, and used it to estimate heterogeneity between the X and Y fragments. This analysis identified considerable heterogeneity and lots of clonal expansion, as expected (Figure 6B). However, when we applied the trained model to the replicates obtained from fragment X, we were disappointed to observe a number of false-positive clonal expansions in both X1 and X2 (Figures 6C,D). Thus, we concluded that since the X1 and X2 replicates were less rich in cells and correlated poorly with the beta-binomial noise model trained on a pair of extra-deep control repertoires (Y1 and Y2), this model must be erroneously identifying statistically significant clonal expansions.

This experiment clearly shows how easy it is to make a mistake in interpreting data pertaining to local immune repertoire clonality, even in an apparently well-controlled experimental setup. Training on replicates of appropriate and comparable depth, both in terms of cell counts and TCR/immunoglobulin cDNA molecule counts, is therefore critical. For identification of immunoglobulin clonal expansions at the mRNA level, which mainly reflect clonality of locally-produced antibodies (5), we recommend training noise models on replicates containing comparable numbers of plasma cells among all samples of interest.

Nevertheless, we observed a prominent difference between the X and Y subsections, and note that the extent of miscorrelation differed significantly between experimental (X1/Y1, X2/Y1, X2/Y1, X2/Y2) but not control (X1/X2, Y1/Y2) samples (Figure 6E). Thus, the proposed approach is relatively stable against additional noise resulting from limited sampling depth when used to estimate the heterogeneity of immune repertoires between tumor sections based on general miscorrelation of clonal frequencies.

## Discussion

It is difficult to measure immune repertoire heterogeneity in terms of relative overlap between repertoires sampled from different parts of the same tumor. Take the example of two tissue sections that initially contain identical repertoires of 100 different T cells where each clone is represented by a single cell. If, in our experimental setup, we were to sample a repertoire of 10 T cells from each section, the overlap would typically comprise 1 TCR variant—i.e., 10% of each sample. If we sampled 50 T cells from each sample, the overlap would grow to 25 variants, or 50% of the repertoire. If we sampled all T cells, the overlap would reach 100%. This simplistic example clearly shows that measurements of repertoire overlaps between tissue sections, T or B cell subpopulations, or time points strongly depend on the depth of repertoire analysis in terms of the number of sampled cells and TCR/immunoglobulin molecules (41). One possible way to analyze relative repertoire overlap across sections is normalization of profiling depth, by downsampling to the same number of analyzed cells or UMI-labeled template TCR or immunoglobulin cDNA/gDNA (39).

Furthermore, a clone present in multiple samples should not be simply defined as being ubiquitously present, since its frequency may differ in terms of order of magnitude. On the other hand, no clone can be defined as non-ubiquitously present, since any given clone could be easily lost in analysis due to sampling limitations.

Prominent cell-to-cell differences in TCR and (especially) immunoglobulin expression levels, inaccuracies in RNA extraction and library preparation, and the stochastic nature of PCR amplification all contribute to degradation of repertoire data quality, and this noise contribution is increased at limited sampling volumes. These considerations dictate that appropriate noise models should be trained on replicates obtained at the level of single-cell suspensions, and that such training should be performed on replicate samples in which T or B cell numbers and expression levels are comparable with those of the samples of interest.

The approach that we propose here addresses both issues, enabling (1) estimation of repertoire heterogeneity across and between tumor tissues, and (2) identification and quantification of locally expanded TCR or immunoglobulin clonotypes. The approach controls for the sampling limitations in a given experimental setup, and allows one to quantitatively judge the extent of repertoire heterogeneity in terms of miscorrelation in clonal frequencies between two samples, as compared to the correlation between control replicates obtained at the level of split single-cell suspensions from the same sample.

This approach can also be used to unequivocally identify the number of locally expanded clonotypes and measure the extent of their expansion. Although one cannot conclude whether a particular T or B cell clone is absent in a tumor section under the proposed conditions, it is possible to determine whether a particular clone is locally expanded to a statistically significant extent. The number and size of such local expansions can be measured and compared between different tumor sections, time points, patients, or tumor subtypes.

Linking tumor (42) and immune heterogeneity is critical since local lymphocyte expansions may correlate with the presence or absence of corresponding immunogenic epitopes, that, in turn, may determine the efficiency or inefficiency of immune response against the whole heterogeneous tumor. Therefore, reliable detection of locally expanded T or B cell clones may have important practical applications.

On the one hand, in contrast to the analysis of averaged bulk repertoire of tumor-infiltrating lymphocytes, identification of locally dominant T or B cell clonal expansions may reveal efficient immune response to particular tumor-associated antigens or neoantigens present in a tumor section (21–23, 28). Therefore, reliable capturing of such local expansions could help to reveal tumor-specific T and B cell clones, facilitating development of adoptive T cell and CAR-T therapeutic approaches. Interestingly, detection of tumor-specific TCRs can be further improved if convergent clones targeting the same epitope are found (43).

Second, T and B cell clonal heterogeneity may reflect the overall heterogeneity of immunogenic targets across the tumor. The T cell pressure can sculpt the antigenicity of tumors escaping from immune control (44). At the same time, tumor heterogeneity may be associated with poor prognosis (45, 46), either due to its higher evolutionary flexibility or antigen “dilution”. The rational way to cope with this intrinsic heterogeneity of permanently evolving tumor is to simultaneously target multiple antigens which distribution across the tumor tissues is non-uniform. Identification and engagement of multiple locally expanded T cell clones from distinct tumor sections could potentially assist in such work.

Along with T cell clonality, B cell expansion was also shown to correlate with response to checkpoint immunotherapy (47, 48). Screening of antibodies reconstituted from circulating plasmablasts of responding patients revealed that many of them bind to non-autologous tumor tissues (11). Hence using B cell clones that are locally expanded before or after therapy could substantially narrow the panel of antibodies with potential reactivity against tumor antigens.

The hopes of today's oncology researchers are, to a great extent, connected with progress in the study of immune repertoires, and the development of methods for the reliable and rapid identification of predictive immune signatures and therapeutically relevant T and B lymphocyte clones. The ability to reliably judge the degree of heterogeneity of immune repertoires and capture local clonal expansions will be an important component of these efforts, and we thus hope that our work will become a useful advance along this yellow brick road.

## Methods

### Murine Tumor Model

Experiments were carried out on C57BL/6 female mice aged 3–5 months. Tumors were generated by subcutaneous (s.c.) injection of 10^5^ B16F0 cancer cells in 300 μL PBS into the left flank. B16F0 melanoma cells were grown in DMEM medium supplemented with 10% fetal bovine serum (FBS), 0.06% L-glutamine, 50 units/mL penicillin and 50 μg/mL streptomycin. Cells were incubated at 37°C and 5% CO_2_, and passaged 2–3 times per week. Right before injection, cells were detached by trypsin, counted, and resuspended at a final concentration of 10^6^ cell in 3 mL PBS. After 3 weeks, ~60% of tumors reached a size of ~1 cm. Mice with linear tumor size ranging from 0.5 to 1.2 cm were sacrificed with isoflurane (Esteve, Italy) in a single day and tumors were surgically removed and prepared for further analysis. All experiments on animals were carried out in accordance with the National Institutes of Health (NIH) Guide for the Care and Use of Laboratory Animals (NIH Publications No. 8023, revised 1978). The experimental protocol was approved by the Ethical Committee of the Privolzhsky Research Medical University Academy, Russia (EC #6, granted April 17, 2019).

### Mouse Melanoma Resection and Lymphocyte Isolation

Freshly-excised tumors were thoroughly cleaned from the outer tumor capsule and either cut into pieces or processed as a whole. For lymphocyte isolation, excised tumor nodules or tumor fragments were homogenized with a gentleMACS dissociator (Miltenyi Biotec, Germany) and incubated in 1–2 mL RPMI medium supplemented with 417 μg/mL Liberase TL (Roche, Germany) and 10 μg/mL DNase I (Roche, Germany) for 30 min at 37°C in a shaker. After dissociation, cell suspensions were passed through a 70 μm cell strainer and washed twice with 5 mL of incubation buffer (PBS pH 7.2, containing 0.5% bovine serum albumin and 2 mM EDTA). Cell pellets were resuspended in 100 μL of incubation buffer with the following antibodies (2 μL each): CD45-PerCP/Cy5.5 (Clone 30-F11, BioLegend), CD3-APC (Clone 145-2C11, BioLegend), CD4-V450 (Clone RM4-5, BD Biosciences), CD8a-APC/Cy7 (Clone 53.6–7, BioLegend), CD19-PE/Cy7 (Clone 6D5, BioLegend). Four hundred microliter of incubation buffer was added after 45–120 min staining at 4°C. CD3^+^CD4^+^ and CD3^+^CD8^+^ subsets were sorted with a FACSAria III cell sorter (BD Biosciences) using an 85 μm nozzle directly into 200 μL RLT cell lysis buffer (Qiagen). After sorting, the samples were vortexed and then maintained at room temperature for 10 min to ensure cell lysis before storing at −20°C.

### Immunohistochemical Staining and Analysis

Immunohistochemical (IHC) analysis of mice tumors was done using Zn-fixed paraffin-embedded tissue slices prepared as described previously (49). Briefly, a 2–3-mm-thick piece was cut from the side of the tumor with the cutting plane perpendicular to the skin, and then transferred into formalin-free Zn fixative (BD Bioscience) for 48 h at 22°C. After fixation, tumor samples were washed twice with PBS, dehydrated with eight consecutive isopropyl alcohol (Isoprep; Biovitrum, Russia) baths at 22°C for 1 h each, followed by 1 h in Histomix paraffin (Histomix, Biovitrum) at 57°C, end embedded into fresh paraffin. 4-μm-thick tissue slices were cut parallel to the cut-off plane using a RM2245 microtome (Leica, Germany) and transferred to Superfrost Plus Gold Slides (Thermo Scientific). For staining, slices were deparaffinized by 2 min incubation in Xylol (twice), 96% ethanol (twice), 70% ethanol, and ddH_2_O. These slides were then processed further either for hematoxylin and eosin (H&E) staining or for multicolor IHC staining.

For IHC staining, slides were dried and marked with a hydrophobic barrier pen (Diagnostic BioSystems), then washed in TBST buffer (Cell Marque, USA) for 5 min and blocked with 5 mg/mL casein (PerkinElmer) in TBST for 30 min at RT. Pre-dissolved primary and secondary antibodies or streptavidin were added directly into blocking solution in a dropwise manner. The following primary antibodies, dilutions, and incubation conditions were used: 1:500 anti-CD4 clone EPR19514 (Abcam), overnight at 4°C; 1:250 biotinylated anti-CD8 clone 53.6–7 (BioLegend), 2 h at RT; and 1:250 biotinylated anti-CD45R/B220 clone RA3-6B2 (BioLegend), 2 h at RT. After staining, slides were washed twice in TBST for 5 min, blocked with 5 mg/mL casein in TBST for 10 min at RT, and then incubated for 1 h at RT with 1:1,000 HRP-labeled secondary antibodies (PerkinElmer) or 1:200 HRP-labeled streptavidin (PerkinElmer) in blocking solution. After secondary antibodies the slides were then washed twice in TBST for 5 min and incubated with a 1:75 dilution of Opal TSA reagent (PerkinElmer) in amplification diluent (PerkinElmer) for 10 min at RT. Primary antibodies and Opal reagents were used in the following order and combinations: anti-CD4 with Opal520, anti-CD8 with Opal570, and anti-CD45R/B220 with Opal650. Opal-treated slides were washed once with TBST for 5 min and processed for antibody stripping and re-staining. For antibody stripping, slices were submerged for 10 min in 0.1 M glycine solution (pH 10.0) and then washed once in TBST before re-staining. After antibody staining was complete, 3 μg/mL DAPI was applied in PBS buffer for 10 min, followed by a 5 min wash in ddH_2_O. Slices were immersed in glycerol and covered with a coverslip that was fixed with a nail polish. Fluorescent images of whole slices were acquired with a 10X objective (NA0.45) on an Eclipse Ti microscope (Nikon, Japan) with an Andor Neo high dynamic range sCMOS camera (ANDOR, UK). The following excitation and emission filters were employed: 377/50 and 447/60 for DAPI, 480/40 and 535/50 for Opal520, 525/50 and 600/50 for Opal570, and 620/60 and 705/72 for Opal650. Whole slices were scanned in multi-point acquisition mode with NIS Elements software (Nikon, Japan) with 10–20% image overlap. To obtain multicolor images of the whole slice, single-color images were stitched with the MIST plugin for ImageJ (50) and overlaid.

H&E staining was done with Mayer's hematoxylin and eosin (Biovitrum, Russia). Whole tissue slices were scanned with a DM2500 microscope (Leica) equipped with motorized stage (Märzhäuser Wetzlar, Germany) using a 20X objective (NA0.4) and LAS software. Images were stitched with the MIST plugin.

### RNA Isolation From Human Tumor Samples

Melanoma material was obtained at N.N. Blokhin Russian Cancer Research Center (Moscow, Russia) from 81 y.o. female patient with stage IIID melanoma (from metachronic inguinal lymph node metastasis). Samples of primary colorectal tumor were collected at Volga District Medical Center (Nizhniy Novgorod, Russia) from a 54 y.o. patient with a stage 4 tumor and metastases in liver and lungs. This study was carried out in accordance with ICH-GCP. The protocol was approved by the Ethical Committees of the Volga District Medical Center under Federal Medical and Biological Agency and of N. N. Blokhin Russian Cancer Research Center of Ministry of Health of the Russian Federation. All specimens were taken with patients' written informed consent.

For analysis of BCR heterogeneity in human metastatic melanoma, an excised lymph node that was fully invaded by tumor metastasis was first cleaned of surrounding connective and fatty tissue. The tumor was cut into two pieces, which were then processed separately. tumor pieces were cut into smaller pieces and incubated in 1–2 mL RPMI medium supplemented with 417 μg/mL Liberase TL (Roche, Germany) and 10 μg/mL DNase I (Roche, Germany) for 30 min at 37°C in 5% CO_2_. Samples were then homogenized with a gentleMACS dissociator (Miltenyi Biotec, Germany). After dissociation, cell suspensions were passed through a 70 μm cell strainer and washed twice with 50 mL of PBS (pH 7.2) containing 0.5% bovine serum albumin and 2 mM EDTA. The pellets were resuspended in PBS at a concentration of 5 × 10^5^ cells/100 μL. Two replicates of 50 μl cell suspension was lysed in 350 μl of RLT cell lysis. Colorectal tumor sections were processed similarly with minor modifications (e.g., homogenization with gentleMACS was performed before incubation in Liberase TL and DNase I). Two 500–1,000 μm^3^ colorectal tumor sections were taken from distant regions of the primary tumor, which were ~3 cm apart.

### Immune Repertoire Analysis

TCR repertoire profiling was performed with a unique molecular identifier (UMI)-based 5′RACE kit (MiLaboratory), similar to described in Egorov et al. (51). Obtained TCR beta CDR3 clonesets are deposited on Figshare (https://figshare.com/s/98d3d72668acabe91a64). Immunoglobulin heavy chain CDR3 repertoire profiling was performed with UMI-based 5′RACE as described in Turchaninova et al. (40). Sequencing was performed on an Illumina MiSeq in 150 + 150 nt paired-end mode. UMI-based clustering of raw sequencing reads was performed with MiNNN software (https://github.com/milaboratory/minnn). CDR3 repertoire extraction was performed using MiXCR (5, 52, 53). Analysis of differential expression between samples was performed as suggested by Rytlewski et al. (32). Only clonotypes having three UMIs or more were used for differential expansion testing.

## Data Availability Statement

The datasets generated for this study can be found on Figshare (https://figshare.com/s/98d3d72668acabe91a64).

## Ethics Statement

The studies involving human participants were reviewed and approved by the local ethical committee. The patients/participants provided their written informed consent to participate in this study. The animal study was reviewed and approved by the Ethical Committee of the Privolzhsky Research Medical University Academy, Russia (EC #6, granted April 17, 2019).

## Author Contributions

DY, LV, ES, IAS, TN, AI, and EB worked with tumor material. MP, TN, DZ, MK, MI, DC, and GS worked on data analysis. ES and EB worked on immunoglobulin profiling. EB, DZ and GS worked on immunohistochemistry. VZ and IVS provided and characterized tumor material. DC, EZ, and GS worked on the manuscript and supervised the study.

## Conflict of Interest

The authors declare that the research was conducted in the absence of any commercial or financial relationships that could be construed as a potential conflict of interest.
